# Patient Satisfaction with Telehealth in Rural Settings: A Systematic Review

**DOI:** 10.5195/ijt.2020.6303

**Published:** 2020-12-08

**Authors:** Loriana C. Harkey, Sadie M. Jung, Elizabeth R. Newton, Angela Patterson

**Affiliations:** 1 School of Pharmacy and Health Professions Department of Occupational Therapy, Creighton University, Omaha, Nebraska, USA

**Keywords:** Client satisfaction, Occupational therapy, Patient preference, Patient satisfaction, Physical therapy, Remote, Rural, Speech-language therapy, Telehealth, Telemedicine

## Abstract

Telehealth provides health care services to clients through telecommunications. Rehabilitation services such as occupational therapy, physical therapy, and speech-language therapy can be delivered via telehealth. The aim of this study was to evaluate patients' reports of their satisfaction with telehealth compared to standard in-person therapy for patients living in rural areas. Four databases were utilized for this systematic review. The following words were searched: telehealth, rural, and patient satisfaction. Abstract searches identified 251 articles, and 55 were read in full text. Four articles met inclusion criteria. There was high satisfaction for patients in all studies regarding the use of telehealth. Findings showed that overall, telehealth supported increased rates of patient satisfaction for OT, PT, and SLP services delivered to rural communities.

The World Bank (2018) reports that 45.17% of the world population live in remote areas, accounting for an ample amount of the worldwide population, thus culminating in a vast shortage of health care services delivered to individuals in these populations. Rural is defined as “all population, housing, and territory not included within an urban area” (Health Resources and Services Administration, 2018, para. 2). As of 2010, there were significantly less health care providers across several professions serving individuals living in rural areas (World Bank, 2018). In 2016, 43.2% of the Chinese population was reported to be living in rural areas (Trading Economics, 2019a). Additionally, 66.46% of the population in India was reported to live in rural locations – 2/3 of the entire population (Trading Economics, 2019b). In Australia, 7 million people live in rural or remote areas in the country, making up 29% of their population (Australian Institute of Health and Welfare, 2018). The U.S. Census Bureau reported that in the United States, 59.5 million people, or 19.3% of the population, live in rural areas (Solovieva & Walls, 2014). In the U.S., there were 13.1 physicians/surgeons per 10K for rural areas compared to 31.2 physicians/surgeons per 10K for urban areas, with a ratio of 0.42 per capita of rural to urban physicians/surgeons (U.S. Department of Health and Human Services, n.d.).

Health discrepancies in rural populations compared to urban as a result of lack of health care services include the following: higher rates of chronic disease, damaging behaviors such as poor dental hygiene and smoking, and greater risk for mental illness and substance abuse, all of which can contribute to poor overall health and quality of life (Van Dis, 2002). Evidence reveals the significant need around the world for more efficient forms of health care delivery to provide care to those who have little to no access to health care. For occupational therapy, there were 2.0 occupational therapists per 10K for rural areas compared to 3.0 occupational therapists per 10K for urban areas, with a ratio of 0.66 per capita of rural to urban occupational therapists (U.S. Department of Health and Human Services, n.d.). In physical therapy, there were 4.4 physical therapists per 10K for rural areas compared to 6.5 physical therapists per 10K for urban areas, with a ratio of 0.67 per capita of rural to urban physical therapists (U.S. Department of Health and Human Services, n.d.).

Telehealth is the remote delivery of health-related services through telecommunication technology to clients for diagnoses, treatment, and prevention of disease and injuries, research and evaluation, and continuing education for health care providers (Koivunen & Saranto, 2018; Bagchi et al., 2018; Jacobs et al., 2015). There are many different identified categories of telehealth, including but not limited to telecardiology, telemedicine, and telerehabilitation. Telehealth is often referred to as telemedicine, which is defined as using real-time audio-video communication between health care providers and patients, storing data for later interpretation, and using remote patient monitoring tools, such as home blood pressure monitors (Balestra, 2018). The American Occupational Therapy Association ([AOTA], 2010) defines telerehabilitation as the “application of evaluation, preventative, diagnostic, and therapeutic services via two-way or multi-point interactive telecommunication technology” (p. S92). However, AOTA endorsed the term “telehealth” in 2013, as it is an all-encompassing term that accurately represents the scope of occupational therapy practice and is more widely used in federal policy (AOTA, 2013, 2018). Similarly, the American Physical Therapy Association also endorsed the term “telehealth” over “telerehabilitation” (American Physical Therapy Association, 2019). For the purposes of this paper, we will refer to telerehabilitation as “telehealth.”

Telehealth can bring necessary physical and occupational therapy services to rural areas. Telehealth is important because it provides therapy to underserved populations living in remote areas that are otherwise unavailable for clients (AOTA, 2018). Rehabilitation services provided via telehealth, including occupational and physical therapy, pose a solution to the health care disparity individuals in rural areas face (Betts et al., 2018).

There are many benefits to providing rehabilitation via a telehealth service delivery model. In 2018, over 90% of health care executives in the United States stated their organizations were currently implementing more telehealth practices, which will provide an alternative to health care services outside of the standard in-person practice setting for an estimated 7 million patients (Flanagan, 2018). An important theme that various studies reported regarding telehealth was reduced mileage and money saved with the availability of telehealth for rehabilitation in remote locations worldwide. Telehealth greatly reduces the cost of therapy for health care companies and for organizations such as the U.S. Department of Veterans Affairs (VA) (Desko & Nazario, 2014). There was an overall $2,317.51 saved for the VA and a 9,000-mile reduction in patient travel as a result of the VA telehealth pain management clinic, which allowed patients to be treated for pain and prescribed medication via telehealth videoconferencing (Desko & Nazario, 2014). Additionally, physical therapy treatments given via the Rural Veteran TeleRehabilitation Initiative saved 3000-5000 miles in travel, a total of 50 hours in driving, and saved between $1,150-1,330 per client in travel expenses (Levy et al., 2015). The Burns Telehealth Service also reduced costs for patients and offered assistance in determining if patients should be admitted into acute care, outpatient, or stay at home while using videoconference, photos, and telephone for wound management (McWilliams et al., 2016).

There are contradictory findings regarding the effectiveness of telehealth. While access to a higher quantity and quality of health care services is assumingly desired, it is necessary to study patient satisfaction with telehealth services provided to individuals living in rural areas. Patient satisfaction leads to returning customers, improved patient retention, profitability, an increase in money spent on public health, positive clinic outcomes including improved safety, accessibility, comprehensiveness of care, and overall quality of health care (Prakash, 2010; Xesfingi & Vozikis, 2016). Several studies stated low patient satisfaction as an outcome of barriers such as inadequate training on use of telehealth technologies, internet connection issues, privacy concerns, or patient preference for in-person communication (Bagchi et al., 2018; Balestra, 2018; Breeden, 2016; Cary et al., 2016; Chedid et al., 2013; Hall et al., 2013; Jacobs et al., 2015; Lade et al., 2012; Parker et al., 2018; Russell et al., 2010). While two studies stated that patients preferred telehealth or that the benefits of telehealth outweighed the barriers (Desko & Nazario, 2014; Levy et al., 2015), others indicated that patients still preferred standard in-person therapy despite high patient satisfaction with telehealth services (Lade et al., 2012; Russell et al., 2010). Furthermore, a few studies indicated either no significant difference in patient satisfaction between telehealth and standard in-person therapy, or stated that patients rated the two forms of therapy as equally effective (Cady & Finkelstein, 2014; Linder et al., 2015; Worboys et al., 2018).

Overall, there is a need to systematically assess patient satisfaction with rehabilitation delivered via a telehealth service delivery model. The aim of this study was to complete a systematic review to evaluate patients' reports of their satisfaction with telehealth for therapy compared to standard in-person therapy for patients living in rural areas.

## METHODS

### SEARCH PROCEDURES

This study was a systematic review. The databases that were utilized for our comprehensive search method for sources obtained in this study were as follows: CINAHL, MEDLINE, PsychINFO, and Cochrane. All databases were accessed via a university library portal. The search strategy included searching the following words on all the databases: telehealth, rural, patient satisfaction. We expanded each term into further search terms such as telerehabilitation under telehealth and patient preference under patient satisfaction to ensure all relevant articles were included. We did a systematic search using similar search terms in each database.

The following term was searched across all databases: telemedicine. Additionally, we used these specific terms for certain databases based upon the suggested search terms.

Medline: (telemedicine OR telerehabilitation OR remote consultation OR teleradiology OR telepathology OR distance counseling) AND (patient satisfaction OR patient preference OR personal satisfaction) AND (rural population OR rural health services OR rural health OR rural nursing).

CINAHL: (telehealth OR telemedicine OR telenursing OR telepsychiatry) AND (patient satisfaction OR patient preference) AND rural areas.

PsychINFO: (telemedicine OR teleconferencing OR online therapy OR teleconsultation OR telepsychiatry OR telepsychology OR telerehabilitation) AND (client satisfaction OR consumer satisfaction OR job satisfaction OR marital satisfaction OR need satisfaction OR relationship satisfaction OR role satisfaction OR sexual satisfaction OR client attitudes OR attitudes OR client satisfaction OR therapist selection OR treatment barriers OR treatment compliance) AND rural environments.

Cochrane: (telehealth OR telerehabilitation OR telemedicine OR teleconferencing OR telepsychiatry OR telepsychology OR digital interventions OR telecommunications OR telenursing OR remote consultation) AND (patient satisfaction OR client satisfaction OR patient preference OR consumer satisfaction) AND (rural OR rural health OR rural areas OR rural environments).

After completing the database searches, we applied the following limiters to all databases: written in English and published between 2009-2019. The following paragraph reports the number of search results and the number of articles remaining after duplicates were removed via a joint RefWorks account.

Medline: 101 articles. After duplication removal: 96 articles

CINAHL: 39 articles. After duplication removal: 27 articles

PsychINFO: 22 articles. After duplication removal: 16 articles

Cochrane: 88 articles. After duplication removal: 87 articles

Abstracts from articles of all databases were then independently reviewed by co-authors.

Additional article abstracts were then hand-searched on February 12, 2020 from *The International Journal of Telerehabilitation* (IJT) and *The Occupational Therapy Journal of Research* for the year of 2019. One article was found in IJT and the full text was screened for eligibility.

### INCLUSION CRITERIA

**Figure 1:**
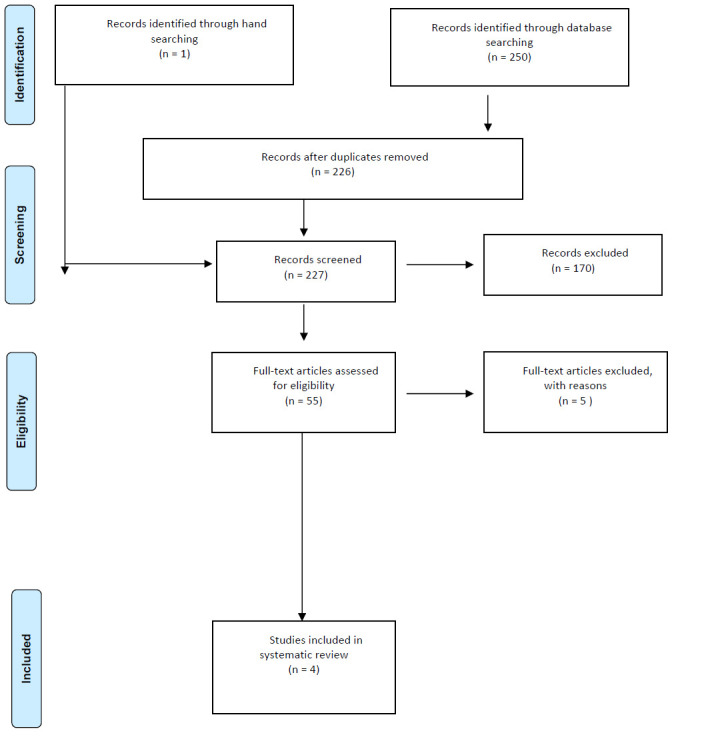
PRISMA Flow Diagram

*Note.* PRISMA flow diagram showing search and selection process that yielded the final four articles.

Eligibility criteria followed the research flow of information procedure (Figure 1), by employing specific terms defined in the search section of the proposal. Inclusion criteria for the study included year of publication, language of publication, study design, and type of article. Study designs included were Levels of Evidence I-V according to Sackett et al. (1996). Previous systematic reviews on telehealth were reviewed to ensure all appropriate articles were accounted for. All articles were peer reviewed empirical research articles. All studies needed to include occupational therapy, physical therapy, and/or speech-language therapy. Relevant articles that matched the inclusion criteria were added to a Microsoft Word document in an abstract matrix format following the evidence template preferred by AOTA (2017). Articles were used if they were fully accessible by the team members via online database access or requested through an inter-library loan.

### EXCLUSION CRITERIA

Articles published prior to 2009 were excluded. Articles that were unpublished trials, editorials, special collections, clinical answers, or other reviews were excluded from this study. Articles that did not pertain to the inclusion criteria were excluded.

### REVIEW PARAMETERS

Upon completing the search for articles, we conducted a study selection process as described in the following sections. A list of all the potential sources utilized in the systematic review were stored on a joint RefWorks account, and all sources were shared and saved onto a joint Microsoft SharePoint folder. The folder functioned as a way for co-authors to access all articles in one place and were accessible from any device. Additionally, a shared document of all the abstracts from the search process was created using a joint Microsoft Word document, accessible via Microsoft SharePoint, and was used to evaluate abstracts during the review process. Each of the co-authors reviewed the abstracts of each article individually and determined if an article should be excluded, included, or reviewed further if the abstract did not have enough information to determine eligibility. Co-authors then met and discussed abstract inclusion, exclusion, and abstracts requiring further review. Abstract inclusion, exclusion, and further review was determined by a 2/3 majority vote. First, articles that required further review were individually read in full text. Second, co-authors met and discussed which articles to eliminate based on inclusion criteria during this second round of reviews. Co-authors formed a final list of articles that still required further review. Each co-author filled out a data extraction form individually for each of the selected articles in the final list to identify similarities in reason for inclusion or exclusion among co-authors (Appendix). Third, co-authors met and discussed article inclusion or exclusion based on the completed data extraction forms from each co-author. Finally, article inclusion or exclusion was determined by a 2/3 majority vote.

### DATA COLLECTION (DATA EXTRACTION FORM FOR SELECTED ARTICLES)

Each co-author filled out a data extraction form individually for each selected article that was included after the full text article review. The data extraction form included the following items: participants, method of subject selection, method of group assignment, study design, blinding, type of intervention, intent to treat analysis, outcomes assessments, compliance, match of interventions and controls, baseline similarity between groups, and patient satisfaction. Co-authors followed the inclusion criteria to identify inter-judge agreement for each included article in this study (Appendix). Co-authors used a 2/3 majority vote to determine which articles to include in the study.

The patient satisfaction section identified factors within the studies that contributed to patient satisfaction (Appendix). Patient satisfaction factors were marked on the list and further explained to synthesize information and provide overall findings of the research regarding patient satisfaction of telehealth in rural areas. Access to this Microsoft SharePoint was only granted to co-authors and involved faculty.

## RESULTS

The database and reference list searches were conducted between November 7, 2019 and December 5, 2019 and yielded a total of 250 articles. Duplications were removed on December 5, 2019 leaving a total of 226 articles. Additionally, one article was found through hand-searching, resulting in a final total of 227 articles. During the article abstract screening process, 170 article abstracts that did not meet inclusion criteria were excluded. The remaining 55 articles were reviewed in full-text and 51 articles that did not meet inclusion criteria were excluded. Four articles remained for the systematic review and the data was recorded. Articles in the full-text review were excluded if they did not pertain to rural settings, occupational therapy, physical therapy, or speech-language therapy, multidisciplinary staff involvement, patient satisfaction, or telehealth services of telephone use or videoconferencing. The review process is detailed in the flow diagram in Figure 1.

### STUDY DESIGN

The four included studies focused on asynchronous and synchronous telehealth service delivery models. Two of the studies utilized a pre-posttest study design (Hall, Gordon, Hulcombe, & Stephens, 2019; Levy, Silverman, Jia, Geiss, & Omura, 2015), one study used a two-group randomized study design (Grogan-Johnson et al., 2010), and the fourth study used a mixed-methods case study design (Sangelaji et al., 2017).

The focused telehealth studies were in rural settings across the United States, Australia, and New Zealand. One study was completed within several schools across four school districts in Ohio (Grogan-Johnson et al., 2010). Another study took place in a general outpatient clinic in remote Queensland, Australia (Hall et al., 2019), while the other took place within the homes of patients in rural North Florida/South Georgia (Levy et al., 2015). The fourth study took place in rural New Zealand within patients' homes (Sangelaji et al., 2017). The studies varied on diagnoses treated and interventions provided; however, all studies utilized patient satisfaction as a primary outcome measure (Grogan-Johnson et al., 2010; Hall et al., 2019; Levy et al., 2015; Sangelaji et al., 2017).

### PARTICIPANTS

Three of the four studies focused on individuals over the age of 15 living in rural areas (Hall et al., 2019; Levy et al., 2015; Sangelaji et al., 2017). One study included four participants between the ages of 56-75 and another included 34 participants between the ages of 4-12 (Grogan-Johnson et al., 2010; Sangelaji et al., 2017). There were no set criteria for age range in the inclusion criteria for this systematic review. All studies had relatively small sample sizes from rural populations with the largest resulting in 69 referrals from medical practitioners or therapists from Queensland Health (Hall et al., 2019).

### TELEHEALTH EQUIPMENT

Technology required to perform telehealth services with patients included synchronous videoconferencing, telephone communication, and asynchronous telehealth websites with videos. Videoconferencing was utilized as a form of telehealth communication in all four studies (Grogan-Johnson et al., 2010; Hall et al., 2019; Levy et al., 2015; Sangelaji et al., 2017). Sangelaji et al. (2017) also utilized an asynchronous approach via web-based physiotherapy programs sent to their patients where the patients were able to view previously recorded videos of necessary exercises. Additionally, telephone communication was used in two studies to check on patient progress and safety with the programs (Hall et al., 2019), and to alter interventions based on patient report (Sangelaji et al., 2017).

### INTERVENTION TYPE, INTENSITY, AND TARGETS

All four studies utilized telehealth services for various interventions. Within the studies, telehealth and telerehabilitation were used interchangeably but refer to the same practice of rehabilitation via telehealth systems. In the study by Grogan-Johnson et al. (2010), speech-language pathologists treated students for articulation, language, and/or fluency disorders. Speech-language therapy interventions consisted of four months of telepractice and four months of conventional therapy for speech-language pathology in the schools with e-helpers for technology problems (Grogan-Johnson et al., 2010). Speech-language pathologists provided on-site therapy to groups of 2-4 students, but most therapy delivered via telehealth was individualized. Physical therapy interventions consisted of physical therapy delivered via an in-home video telehealth program called the Rural Veterans TeleRehabilitation Initiative (RVTRI) (Levy et al., 2015). Participating veterans were enrolled through physiatric mild traumatic brain injury clinics, spinal cord injury/mobility clinics, and general physical therapy clinics (Levy et al., 2015). Physiotherapy and occupational therapy were other forms of rehabilitation provided in two studies (Hall et al., 2019; Sangelaji et al., 2017). In one study, web-based physiotherapy programs were conducted for 12 weeks followed by Blue Prescription (BP) intervention (an intervention targeting behavior change) for patients with multiple sclerosis (Sangelaji et al., 2017). These interventions were delivered by New Zealand registered physiotherapists trained in web-based physiotherapy (WBP). This program consisted of over 200 videos of exercises that participants were asked to complete. Additionally, patients were asked to complete a “digital diary of exercise participation via the internet” (Sangelaji et al., 2017, p. 17). Diaries were available to therapists to alter the participants' programs, observe progress, and “monitor adherence and adverse events” (Sangelaji et al., 2017, p. 17). The therapist and patient were also in contact for support throughout via telephone, email, and videoconferencing. In the study by Hall et al. (2019), telehealth coaching was provided by physiotherapists and occupational therapists utilizing standard videoconferencing units or cameras for compression garment selection, fitting, and monitoring of services for individuals with lymphedema.

### OUTCOME MEASURES

The included studies examined the effectiveness of interventions via telehealth using patient satisfaction as an outcome measure. Overall, a majority of participants across all studies reported positive experiences as a part of their patient satisfaction feedback (Grogan-Johnson et al., 2010; Hall et al., 2019; Levy et al., 2015: Sangelaji et al., 2017). Most participants in two of the studies reported that they would use videoconferencing to receive telehealth services again (Grogan-Johnson et al., 2010; Levy et al., 2015).

For patient dissatisfaction, four of 29 participants in one study reported that they could not see or hear the therapist on the videoconference (Grogan-Johnson et al., 2010), participants from two of 38 sessions reported having poor quality images that did not provide enough clarity to demonstrate or assess the task (Hall et al., 2019), and participants from another study reported that the web-based physiotherapy program over time became ‘boring,' ‘tedious,' and ‘monotonous' (three of four participants), that the activity monitor was ‘very uncomfortable' to wear especially when sleeping (one of four participants) and that they forgot the website password (one of four participants) (Sangelaji et al., 2017). One participant suggested that using the web-based physiotherapy intervention would have been more beneficial at a younger age: “I've had [multiple sclerosis] for 30 years, 10-15 years ago I was a lot more active than I am now” (Sangelaji et al., 2017, p. 19).

For patient satisfaction, one participant stated: “I think the idea is really good especially for rural people” (Sangelaji et al., 2017, p. 19). One study reported “extremely high levels of satisfaction with the providers' personal manner; interactions with providers during the care, privacy, and operation of telehealth equipment; and the audiovisual quality of the equipment” and 92% (23 of 25 participants) stated that they were able to connect with their physical therapist in five minutes or less (Levy et al., 2015, p. 366). This study also reported that all participants stated that they would use telehealth again for medical care (Levy et al., 2015). In another study, 92% of participants (approximately 53 of 58 participants) were satisfied with their experience, 5% were moderately satisfied (approximately three of 58 participants), and 2.5% (approximately 1 of 58 participants) provided no response (Hall et al., 2019). Participants provided more positive than negative feedback regarding patient satisfaction in all four studies.

## DISCUSSION

### FINDINGS

This systematic review investigated patient satisfaction of telehealth services that were delivered through videoconferencing, asynchronous telehealth websites with videos, and telephone communication. All four of the analyzed studies included videoconferencing. The findings of this review revealed that there is high patient satisfaction with telehealth services for occupational therapy, physical therapy or physiotherapy, and speech-language therapy. Most of the participants in all studies reported satisfaction with telehealth or indicated that they would utilize services again (Grogan-Johnson et al., 2010; Hall et al., 2019; Levy et al., 2015; Sangelaji et al., 2017). A few participants across all studies reported dissatisfaction due to poor technological quality or other program difficulties (Grogan-Johnson et al., 2010; Hall et al., 2019; Levy et al., 2015; Sangelaji et al., 2017). The four studies all differed in types of rehabilitation services provided via telehealth, and therefore further studies are necessary for more in-depth reviews of specific telehealth services.

These findings contribute to the benefits of the expansion of telehealth as a service delivery model in rural settings. Because of the high satisfaction ratings related to ease of travel, quality of care, safety, and reduced costs, telehealth services should be utilized to deliver therapy to clients in rural settings that have difficulty accessing healthcare services (Grogan-Johnson et al., 2010; Hall et al., 2019; Levy et al., 2015; Sangelaji et al., 2017). Additionally, most participants across two studies preferred telehealth over standard in-person therapy (Grogan-Johnson et al., 2010; Levy et al., 2015).

Other systematic reviews supported these findings as well. A systematic review on the effects of telehealth in occupational therapy practice found that telehealth can be used as an alternative service delivery model (Hung & Fong, 2019). Hung and Fong (2019) only evaluated the effectiveness of rehabilitation for occupational therapy delivered via telehealth and did not use patient satisfaction as the main outcome measure. They also did not find sufficient evidence that telehealth was more effective than standard in-person services (Hung & Fong, 2019). Another systematic review on synchronous telehealth for musculoskeletal conditions found that telehealth was an effective service delivery model and found it to be comparable to standard practices (Cottrell et al., 2017). Cottrell et al. (2017) found that telehealth was slightly more favorable than standard practice and was equally as effective as standard in-person interventions for the improvement of pain. However, Cottrell et al. (2017) did not assess patient satisfaction or cost. Instead, this systematic review provided insight into patient satisfaction related to many measures such as cost and travel.

In addition to providing evidence that telehealth is a viable option for those living in rural locations, this systematic review has implications for future research. The knowledge gained in this study can be used to advocate for the expansion in telehealth services, especially to rural populations or locations that are difficult to reach. This may allow more patients to access quality health care who otherwise would have difficulty obtaining necessary therapy services.

From a clinical standpoint, this study reveals additional considerations for delivering rehabilitation services via telehealth. In one study, participants were able to contact therapists for questions within five minutes or less, implying that this form of delivery may address patient needs much more quickly than standard in-person therapy (Levy et al., 2015). Because some participants across all studies reported technical difficulties such as poor visual and auditory quality, and/or feelings of violation from cameras in their home, it is necessary for therapists to collaborate with technology support professionals to improve future experiences of patient satisfaction in telehealth delivery (Grogan-Johnson et al., 2010; Hall et al., 2019; Levy et al., 2015; Sangelaji et al., 2017).

For telehealth services to be accessible to patients, proper internet access is required along with certain bandwidth recommendations for successful administration of therapy (Tan et al., 2014). Such technology is available, as identified by Tan et al. (2014), however, it is likely many therapy practices are unaware of these possibilities or do not have technology professionals to serve rural areas. Employing more professional information technology support may greatly improve outreach worldwide. This can potentially benefit developing countries with large populations that lack adequate therapy services. Moreover, health care systems throughout the world would benefit from using telehealth as a service delivery model during pandemics in order to safely continue to provide therapy services to patients, especially those who may be at risk for infection when traveling to outpatient clinics.

This systematic review also implies the need for further program development for training healthcare professionals in how to properly administer therapy services via telehealth. This training may be a crucial component making rehabilitation practices via telehealth more widely used while retaining high patient satisfaction rates.

Overall, the methods for utilizing telehealth as a service delivery model to provide therapy are effective for rural populations around the world with access to the internet. However, this study presents methodological issues related to a substantial variety in delivery methods, therapy practices, sample sizes, and populations across all studies. This systematic review analyzed three separate disciplines (i.e., OT, PT, SLP) in only four studies, making it difficult to generalize these results to all therapy practices. While three studies had 25+ participants (Grogan-Johnson, et al., 2010; Hall et al., 2019; Levy et al., 2015), one study only had four participants (Sangelaji, et al. 2017). In three out of the four studies, participants were seen virtually for a set therapy time (Grogan-Johnson et al., 2010; Hall et al., 2019; Levy et al., 2015), whereas in the fourth study virtual visits were only discussed on a consultative and adjustment basis, that is when a physiotherapist could alter the provided exercise regime to fit the patient's needs (Sangelaji et al., 2017). Despite these complications, a theme emerged regarding the telehealth service delivery model. Synchronous videoconferencing elicited the most positive feedback from participants across studies compared to all other forms of telehealth (Grogan-Johnson et al., 2010; Hall et al., 2019; Levy et al., 2015; Sangelaji et al., 2017). Thus, future research should consider using synchronous videoconferencing to further analyze its effectiveness and aid in the establishment of telehealth as a more commonly used service delivery model. Specifically, more randomized controlled trials for telehealth in occupational therapy, physical therapy, and speech-language therapy for rural populations are recommended.

### LIMITATIONS

As previously stated, there were some limitations to this systematic review. Only articles published between 2009-2019 and written in English were included in accordance with the inclusion criteria. Articles written in English with research conducted outside of the United States were also included.

This systematic review did not include dissertations, literature reviews, conference abstracts, posters, unpublished papers or trials, white papers, protocols, editorials, special collections, or reviews. For data collection, co-authors used four databases accessible through a university portal to search for articles. In terms of the design of this study, systematic reviews fall under Level I, the highest level of evidence according to the hierarchy established by Sackett et al. (1996). This systematic review resulted in a total of four articles, which is a relatively small sample. It includes two articles with research conducted in the United States, one article with research conducted in New Zealand, and one article with research conducted in Queensland, Australia. With a sample size of four, it is not likely that this systematic review is generalizable to rural U.S. populations or rural populations of New Zealand and Australia. The results of this systematic review indicate a need for more research regarding patient satisfaction with telehealth as a service delivery model for occupational therapy, physical therapy, and speech-language therapy providing service to rural populations.

### FUTURE DIRECTIONS

There is not enough research on patient satisfaction for the use of telehealth as a service delivery model for rural populations in need of occupational therapy, physical therapy, and speech-language therapy. Telehealth is a relatively new service delivery model; however, it is anticipated to become a commonly and widely used service delivery model in the coming years. Two of the four studies in this systematic review indicated the need for more randomized controlled trials and studies with larger sample sizes (Grogan-Johnson et al., 2010; Levy et al., 2015). However, to conduct such research requires the need for more people willing to participate in telehealth studies, receive telehealth services, and offer feedback in the form of patient satisfaction surveys, interviews, or other methods. All studies in this systematic review indicated high levels of patient satisfaction (Grogan-Johnson et al., 2010; Hall et al., 2019; Levy et al., 2015; Sangelaji et al., 2017). This supports the need for more research and increased use of telehealth services for rural populations. While many studies measure patient satisfaction with telehealth, few of those studies were conducted with rural populations. This is problematic because people from rural populations are those who may benefit from this service delivery model the most.

## CONCLUSION

Telehealth is a potential solution to address the need for rural populations to receive rehabilitation services. The results of this systematic review report a remarkably high patient satisfaction rate with telehealth as a service delivery model to provide occupational therapy, physical therapy, and speech-language therapy to rural populations. Across the globe, there is a need for greater access to health care for rural populations. Future research on telehealth should aim to conduct more randomized controlled trials and recruit large numbers of participants resulting in significantly larger sample sizes for study results to be generalizable to larger populations. This systematic review confirms the need for further research regarding patient satisfaction of rural populations with telehealth services.

## APPENDIX DATA EXTRACTION FORM

Data Extraction Form

Study Title _____________________________________________________________________________

Authors _____________________________________________________________________________

Journal ____________________________Year __________ Vol _______ Year ___________

Study Type _____________________________________________________________________________

Methods / Trial Quality

**Table:**

Participants Age (mean, range) Subject inclusion/exclusion criteria				
Method of subject selection				
Method of group assignment (randomization)				
Study design				
Blinding				
Type of Intervention Interventions Control conditions Duration and other protocol information				
Intent to treat analysis				
Outcome assessments				
Match of interventions and controls				
Baseline similarity between groups				
Patient Satisfaction				
	Yes	No	Pro	Con
Safety				
Ease of Travel				
Quality of Care				
Caregiver Efficacy				
In-person vs Virtual Rehabilitation				
Other Please Specify				

*Note.* Data Extraction form for Systematic Reviews adapted from Portney and Watkins, 2009.
